# HEALTH RESILIENCE AND FORTITUDE FACTORS IN THE HRS

**DOI:** 10.1093/geroni/igae098.0206

**Published:** 2024-12-31

**Authors:** Rebecca Allen, Jacqui Smith

**Affiliations:** Chair; Discussant

## Abstract

This symposium presents data from recent waves of the Health and Retirement Study, a nationally representative sample of older adults in the United States, examining environmental transition and health variables as they relate to fortitude. Using stress process models and the Meikirch model of health as theoretical underpinning, these presentations examine fortitude by focusing on 1) transitions in place of residence and loneliness (inverse of fortitude), 2) relationship quality and pain intensity, and 3) sleep disorders, relationship quality, and life satisfaction. The first presenter found that subjective health, transitional threat, and social and community engagement all were significantly related to self-reported loneliness. However, the interaction between engagement and transitional threat did not explain additional variance in the model. The second presenter found that the association between perceived spousal support (personally acquired potential) and well-being significantly decreased when controlling for the intensity of pain (biologically given potential) experienced by the respondent. Similarly, using the Meikirch model of health, the third presenter conducted simple linear regression analyses to assess the relations between sleep disorders, perceived life satisfaction, and perceived spousal relationship quality. Having a sleep disorder significantly predicted life satisfaction and perceived problems in spousal relationships. Thus, perceived stress due to environmental transition and the interplay between biologically given potential and personally acquired potential explain significant variance in fortitude, as measured by loneliness (inversely), well-being, and life satisfaction. The discussion will address strengths and challenges in examining fortitude as a construct of interest in the nationally representative Health and Retirement Study dataset.

